# Sulfated *Cyclocarya paliurus* polysaccharides markedly attenuates inflammation and oxidative damage in lipopolysaccharide-treated macrophage cells and mice

**DOI:** 10.1038/srep40402

**Published:** 2017-01-17

**Authors:** Zhijun Wang, Jianhua Xie, Yujiao Yang, Fan Zhang, Shengnan Wang, Ting Wu, Mingyue Shen, Mingyong Xie

**Affiliations:** 1 State Key Laboratory of Food Science and Technology, Nanchang University, Nanchang 330047, Jiangxi, China

## Abstract

Natural polysaccharides and their modified derivatives are crucial supplements to the prevention of inflammation. This study aimed to evaluate the effect of sulfated modification on the anti-inflammatory and anti-oxidative activities of *Cyclocarya paliurus* polysaccharides (CP). A sulfated CP, S-CP_1–4_ was obtained using chlorosulfonic acid-pyridine method. The chemical components and FT-IR spectrum confirmed that sulfated group was synthesized to the polysaccharide chains successfully. S-CP_1–4_ was found to inhibit nitric oxide production, phagocytic activity and the release of interleukin (IL)-6 and IL-1β in lipopolysaccharide-treated macrophage cells, RAW 264.7. S-CP_1–4_ significantly decreased the secretion of IL-6 and TNF-α and the thymus and spleen indexes, and increased the production of IL-10 in lipopolysaccharide-treated mice. S-CP_1–4_ could better protect the liver by inhibiting the activities of alanine aminotransferase and aspartate aminotransferase, and malondialdehyde level while increasing the superoxide dismutase activity and total anti-oxidative capacity. These results suggested that S-CP_1–4_ may be an effective anti-inflammatory agent, and sulfated modification may be a reliable method for the development of food supplements.

Inflammation is an innate defense mechanism against injury, infection and stress^1^,^2^. Generally, well-organized inflammation has beneficial effects on health by eliminating the invading pathogens and activating the immune system. An excessive inflammatory response, including chronic and acute inflammation, can lead to host cell and tissue injury^2^. The prolonged inflammatory state can increase the risk of developing cardiovascular diseases, hepatitis and cancer^3^,^4^. Modern medicine can be used for the treatment of these diseases with side effects, health food resources is necessary supplement to regulate immune function^5^,^6^.

Lipopolysaccharides (LPS) are produced by the Gram-negative bacterial cell wall. This major endotoxin can induce experimental endotoxemia. The macrophages induced by LPS are valuable and mature cell models to evaluate anti-inflammatory activity of natural products^7^. After treated with LPS, the levels of various cytokines, free radicals and enzymes activities become the primary evaluation indexes for anti-inflammatory activities of polysaccharides, because their production in cells and organs may be changed^8^. Among these compounds, cytokines play an important role in the inflammatory process of macrophages, which can reflect the generation of inflammation; these cytokines include tumor necrosis factor (TNF) -α, interleukin (IL) -1β, IL-6, and IL-10^9^. Nitric oxide (NO) is synthesized in macrophages in response to infection; its level can indicate the degree of inflammation^10^. Reactive oxygen species (ROS), such as superoxides (O^2−^), hydroxyl radical (OH·), and malondialdehyde (MDA), are also generated during inflammation in response to pathogen invasion. The accumulation of these products can mediate a range of disorders, such as atherosclerosis, diabetes, Alzheimer’s disease and cancer^11^.

Nowadays, although steroidal anti-inflammatory drugs as well as aspirin, phenylbutazone have been used in the treatment of inflammation induced by tissue damage, they had side effects in clinical trials. In recent years, many researchers have begun to seek natural compounds due to their anti-inflammatory activities and nontoxicity^12^. It was well known that natural polysaccharides exhibited a wide range of biological activities, especially the antioxidant and immunoregulatory activities^13^,^14^. Several polysaccharides were found to have good anti-inflammatory and hepatic activity^15^,^16^, however, limited by low yield and low activity, it is thus necessary to find a way to strengthen the bioactivities of polysaccharides^17^. Meanwhile, the introduction of sulfated groups has been reported to significantly improve the bioactivity of polysaccharides, including the antioxidant and anti-inflammatory activities^18^,^19^. After sulfated modification, modified polysaccharides could better improve inflammatory condition of macrophages through decreasing phagocytosis activity and NO production^15^. Sulfated fugal polysaccharides from *Antrodia cinnamomea* had a stronger inhibitory effect on TNF-α and IL-6 production as compared with native polysaccharides^19^. Sulfated polysaccharides also contributed to anti-LPS induced oxidative stress in liver, an important immune and digestive organ^20^.

*Cyclocarya paliurus* (Batal.) Iljinskaja has been used as traditional edible food for thousands of years^21^. *C. paliurus*, distributed in most southern provinces such as Hunan, Jiangxi and Zhejiang in China, was reported to be a highly potential new ingredient for food in 2013 by National Health and Family Planning Commission, China^22^. Tea drinks with *C. paliurus* leaves as raw materials have become a popular health product^23^. The extraction process and initial structure of *C. paliurus* polysaccharides (CP) has been reported^24^. CP also has documented bioactivities, such as anti-tumor and antioxidant activities and its promotion of intestinal health^24^,^25^,^26^. To the best of our knowledge, none of the previous studies have focused on the anti-inflammatory activity of CP and its sulfated derivatives.

In the present study, CP and its sulfated derivative (S-CP_1–4_) were isolated and prepared from *C. paliurus*. Their chemical features were analyzed, and their anti-inflammatory activities were evaluated in LPS-infected RAW 264.7 cells and LPS-treated Balb/c mice. The protective effects of S-CP_1–4_ and CP against LPS-mediated liver damage in mice were also investigated in this study.

## Results and Discussion

### Chemical composition and molecular weight

The native CP was obtained from the leaves of *C. paliurus* by water extraction and alcohol precipitation. The sulfated polysaccharide, designated as S-CP_1–4_, was prepared with a chlorosulfonic acid–pyridine ratio of 1:4 (*v:v*).

The total sugar, uronic acid and protein content, as well as the degree of sulfated groups (DS), of CP and S-CP_1–4_ are summarized in Table 1.

The total sugar contents in CP and S-CP_1–4_ were 63.77 ± 1.55% and 42.41 ± 2.55%, respectively. The DS was not detected in CP, but was 0.42 ± 0.04 in S-CP_1–4_. After sulfated modification, the chemical composition, as well as the physical and chemical properties, were changed; these change may have been caused by the polysaccharide degradation that usually occurs in sulfation reactions^27^.

**Figure 1:**
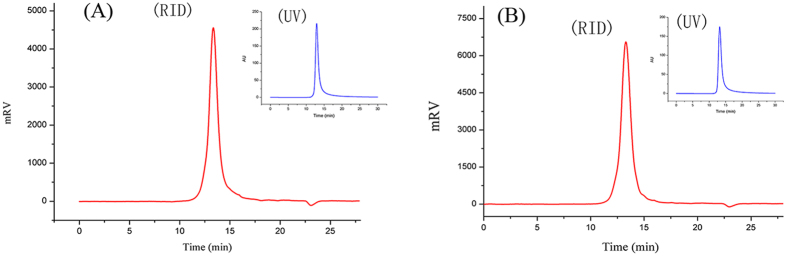
HPGPC chromatogram profiles of CP (**A**) and S-CP_1–4_ (**B**). Each sample was applied onto Ultrahydrogel-500 column with ultrapure water as the eluant at a flow rate of 0.6 mL/min.

Figure 1 shows the homogeneity and molecular weights (*Mw*) distribution of the CP and S-CP_1–4_. CP only showed a single symmetrical peak in the HPGPC chromatogram, indicating that it was a homogeneous polysaccharide. By contrast, S-CP_1–4_ produced a single symmetrically sharper peak than CP. Based on the calibration with the standard dextrans, the average *Mw* of CP and S-CP_1–4_ were estimated to be 1.39 × 10^5^ and 2.12 × 10^5^ Da, respectively. Compared with CP, the *Mw* of S-CP_1–4_ was increased, and its homogeneity was improved. The change of *Mw* can be attributed to the hydrolysis in acid conditions and the addition of a sulfated group during sulfation^28^. Figure 1 also shows small peaks in RID result from HPGPC, which were caused by the different fractions with different *Mw*. In this study, the protein contents of CP and S-CP_1–4_ were 8.23 ± 0.78% and 1.95 ± 0.09%, respectively. According to our previous results, the *Cyclocarya paliurus* polysaccharide was a heteropolysaccharide composed of carbohydrate, protein and uronic acid. The peaks in UV-spectrum were the results of protein bound to the polysaccharides of *Cyclocarya paliurus*. This result was in accordance with the results of our previous study^24^ and other studies in polysaccharides from tea^29^,^30^.

### Infrared spectra

**Figure 2:**
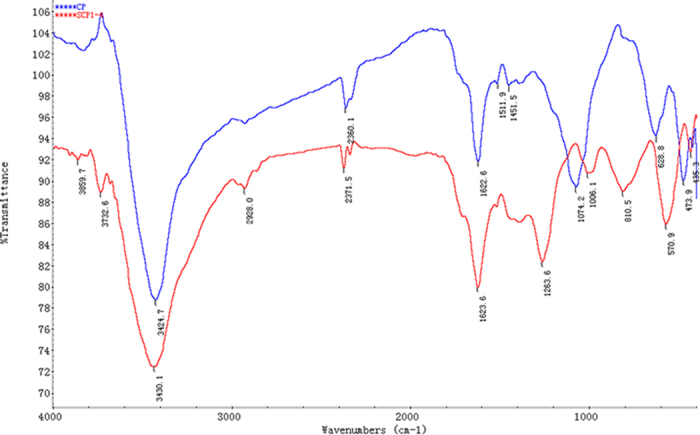
FT-IR spectra of CP (**A**) and S-CP_1–4_ (**B**) between 400 and 4000 cm^−1^.

FT-IR spectroscopy is often used to identify the characteristic absorption peak of polysaccharides. Figure 2 presents the FT-IR spectra of CP and S-CP_1–4_ at a range of 4000–400 cm^−1^ 31. Both polysaccharides had the typical absorption peaks at approximately 3420 cm^−1^ (O–H stretching vibration), 2930 cm^−1^ (C–H stretching vibration) and 2370 cm^−1^ (C–H deviational vibration), thereby confirming the presence of polysaccharides^32^. The absorption peaks at approximately 1620 cm^−1^ corresponded to the N–H deviational vibration and C=O unsymmetrical stretching vibration, further illustrating that the polysaccharides may contain a small amount of protein. The results also confirmed the presence of proteins in the samples (Table 1). The sharp absorption peak at 1259 cm^−1^ (S–O asymmetry stretching vibration) and 813 cm^−1^ (C–O–S symmetry stretching vibration) were assigned to the sulfated group in S-CP_1–4_^33^. The spectral information was similar to the FT-IR spectra of sulfated *Ganoderma atrum* polysaccharides and *Sphallerocarpus* polysaccharides^18^,^34^,^35^.

### Cell viability

**Figure 3:**
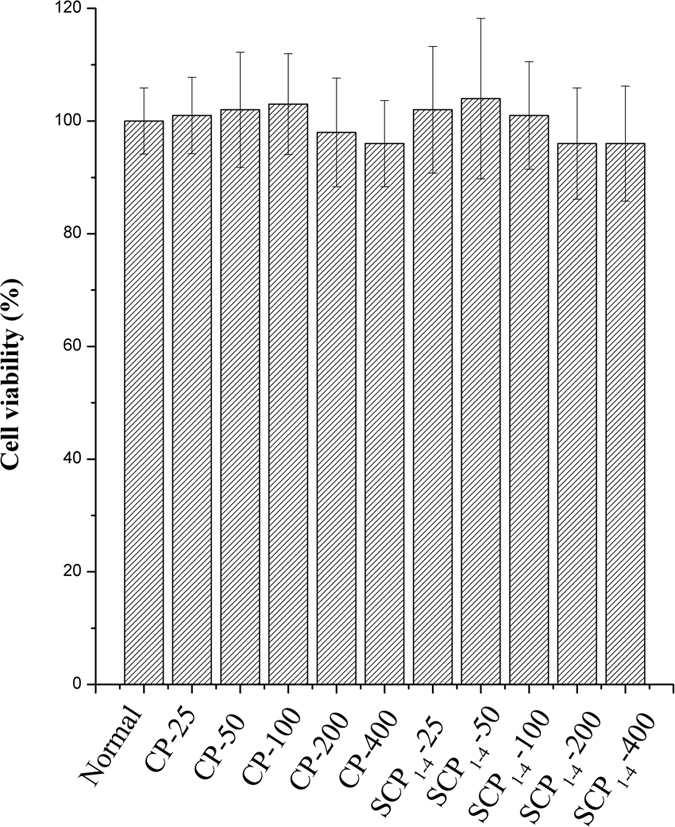
Toxicity test of CP and S-CP_1–4_ on cell viabilities of RAW264.7 cells.

CP and S-CP_1–4_ were selected for bio-assay using RAW264.7 cells. S-CP_1–4_ was a chemical modified derivative. To evaluate the cytotoxicities of CP and its derivative S-CP_1–4_ on RAW264.7 cells, the cells were treated with CP and S-CP_1–4_ at different concentrations (25, 50, 100, 200, 400 μg/mL) and then cultured for 24 h. As shown in Fig. 3, the viability of RAW264.7 macrophages was not significantly influenced by CP and S-CP_1–4_ treatments at 25, 50, 100 and 200 μg/mL. The maximum safe concentration of CP and S-CP_1–4_ was 200 μg/mL. The concentrations of all the polysaccharides used in the study were 25, 50 and 100 μg/mL in order to be easy to compare.

### NO and cytokines in LPS-treated RAW 264.7 cells

NO is involved in signal transduction in the nervous and immune systems. NO could be produced by various immune cells, including macrophages, neutrophils, and natural killer cells^36^. LPS is a bacterial endotoxin that promotes the secretion of pro-inflammtory cytokines and related molecules, including NO, IL-1β, and IL-6, in several cell types. The inhibition of NO overproduction in cells may prevent the occurrence of inflammatory diseases^37^.

**Figure 4:**
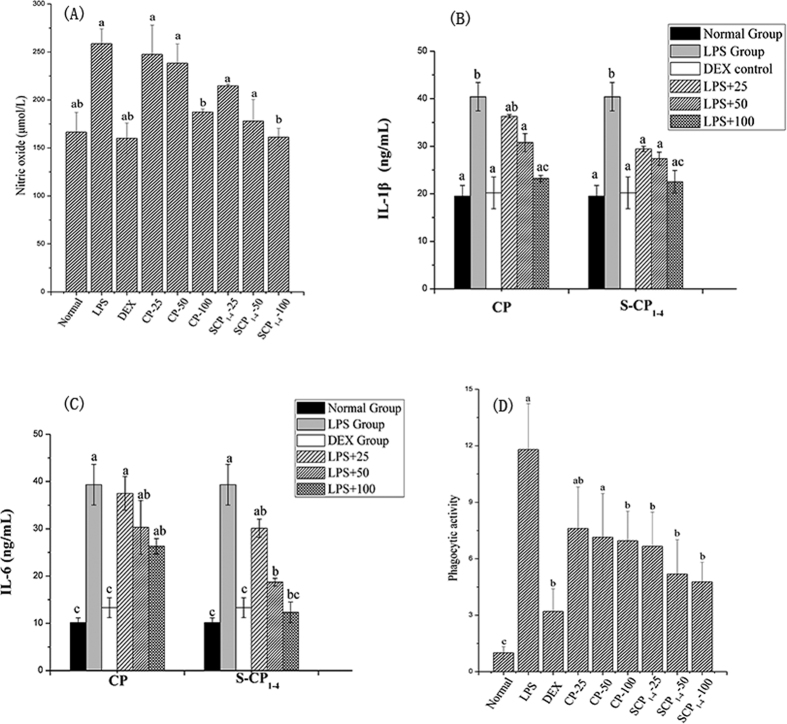
Effect of CP and S-CP_1–4_ on LPS-induced RAW264.7 cells; (**A**) The inhibitory activity of CP and S-CP_1–4_ for NO production in LPS-induced RAW264.7 cells; (**B**) The inhibitory activity of CP and S-CP_1–4_ for IL-1β in LPS-induced RAW264.7 cells; (**C**) The inhibitory activity of CP and S-CP_1–4_ for IL-6 in LPS-induced RAW264.7 cells; (**D**) The inhibitory activity of CP and S-CP_1–4_ for phagocytic activity in LPS-induced RAW264.7 cells; Values are expressed as mean ± SD (n = 6). Data marked without the same letters (a–d) differ significantly (*P* < *0.05*).

As shown in Fig. 4A, LPS stimulation at 1 μg/mL for 24 h caused a massive increase in the NO production compared with the normal group. CP and S-CP_1–4_ treatment could suppress NO production in a dose-dependent manner, whereas CP-100 exhibited the strongest inhibitory activity. Luo *et al*. found that *Astragalus* polysaccharide significantly inhibited NO production in LPS-treated microglial cells^38^.

Consistent with the inhibitory effects on NO production, a dose-dependent suppression of IL-1β and IL-6 production by CP and S-CP_1–4_ was also observed. As shown in Fig. 4B and C, the LPS treatment significantly increased IL-1β and IL-6 production compared with the normal group (*P* < *0.05*). The addition of CP and S-CP_1–4_ dramatically down-regulated IL-1β and IL-6 production (*P* < *0.05*). S-CP_1–4_ was more effective at a concentration of 100 μg/mL. Wang *et al*. reported that the sulfated modification of *Astragalus* polysaccharide enhanced the anti-inflammatory activity by down-regulating TNF-α and IL-1β production in Caco2 cells^39^. These results suggested that sulfated modification could improve the anti-inflammatory activity by limiting NO production as well as the IL-1β and IL-6 secretion.

### Phagocytosis in LPS-treated RAW 264.7 cells

The phagocytic ability of macrophages was measured by the neutral red uptake. One of the most useful indicators of macrophage activation would be enhanced phagocytic activity, which represents a vital step in the immunological defense system^40^. The inhibition levels of phagocytic activity at different concentrations of CP and S-CP_1–4_ in the LPS-treated RAW 264.7 cells are shown in Fig. 4D. Given that 1 μg/mL LPS stimulation significantly increased the phagocytosis activity of RAW 264.7 cells, these cells were activated and the inflammation model was successfully established. The addition of CP at different concentrations decreased the phagocytic activity in LPS-treated RAW 264.7 cells in a dose-dependent manner. Compared with the CP group, the S-CP_1–4_ group showed a stronger inhibitory effect, especially at the highest concentration (100 μg/mL). The phagocytosis of macrophages is a double-edged sword; the excessive activation of macrophages usually causes inflammation and organ damage. The native and sulfated polysaccharides exhibited inhibitory activities on the phagocytic index, and sulfated modification enhanced the inhibition effect.

### Visceral index and liver function in LPS-treated mice

**Figure 5:**
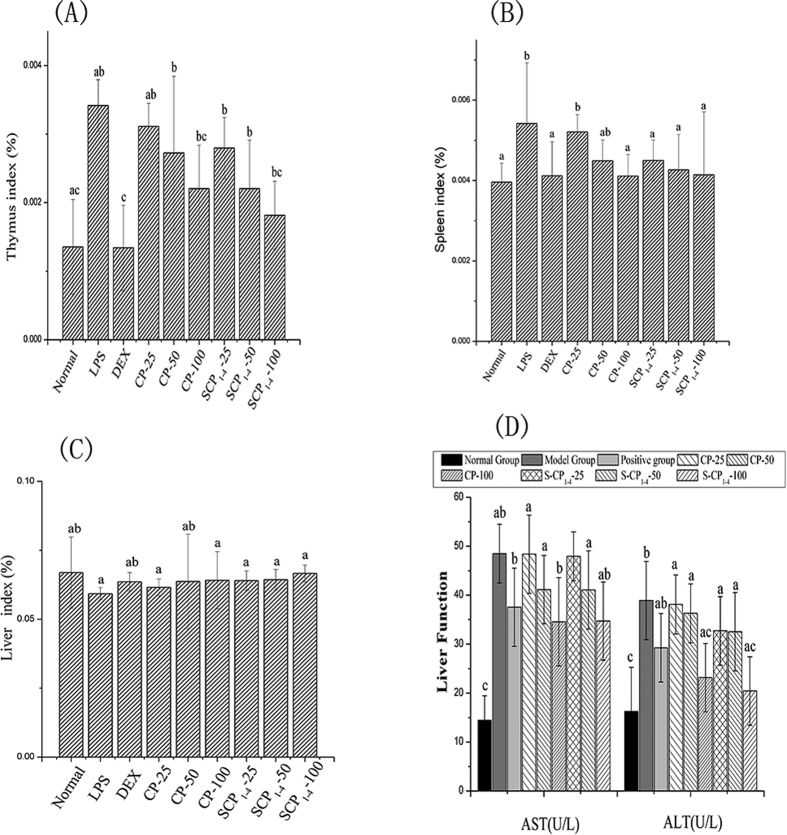
Effect of CP and S-CP_1–4_ on LPS-induced mice; (**A**) The inhibitory activity of CP and S-CP_1–4_ for thymus index in LPS-induced mice; (**B**) The inhibitory activity of CP and S-CP_1–4_ for spleen index in LPS-induced mice; (**C**) The inhibitory activity of CP and S-CP_1–4_ for liver index in LPS-induced mice; (**D**) The inhibitory activity of CP and S-CP_1–4_ for ALT and AST in LPS-induced mice; Values are expressed as mean ± SD (n = 6). Data marked withdifferent letters (a–d) differ significantly (*P* < *0.05*).

The thymus and spleen are important immune organs; their mass indexes could reflect the levels of inflammation. The thymus and spleen indexes increased in the LPS-treated group compared with the untreated control group, indicating that the celiac inflammation model was successfully established. As the positive control, DEX can significantly reduce the degree of inflammation almost close to the normal group. All the polysaccharide treatments decreased the thymus and spleen indexes in a dose-dependent manner. For the thymus index, S-CP_1–4_ at a concentration of 100 μg/mL produced the strongest inhibitory activity. The same result was observed for the spleen index. As shown in Fig. 5C, the liver index was decreased by LPS compared with the normal group. However, the liver index increased in the CP and S-CP_1–4_ treated LPS groups as compared with the LPS group, especially in mice treated with S-CP_1–4_ at the dose of 100 mg/kg (*P* < 0.05). These results showed that the liver disease induced by LPS was alleviated by CP and its sulfated counterpart.

The liver plays an important role in immune regulation, alanine aminotransferase (ALT) and aspartate aminotransferase (AST) are two biomarkers of hepatocellular injury in patients with some degree of hepatic disorder. As shown in Fig. 5D, the ALT and AST activities in the serum were significantly higher than those of the normal group, indicating that the liver cells were damaged and ruptured. Treatments with 25, 50, and 100 mg/kg BW of CP and S-CP_1–4_ decreased the activities of ALT and AST, with 100 mg/kg BW S-CP_1–4_ being the most effective in liver protection.

The increased thymus and spleen indexes showed that intraperitoneally injected LPS could induce an immune response, suggesting the successful establishment of a murine inflammation model induced by LPS (0.5 mg/kg). In this study, CP inhibited the LPS-induced inflammation to protect the immune organs, whereas sulfated modification enhanced its activity. The underlying mechanisms could be a result of the immunoregulatory activity of polysaccharides. Several natural sulfated polysaccharides have strong immunological competence^41^. Sulfated modification has become a focus of growing interest in chemical modification. Wang *et al*. found that the addition of a sulfated group enhanced the immunity-enhancing activity of *Lycium barbarum* polysaccharides by promoting lymphocyte proliferation and enhancing serum antibody titer^42^. Jiang *et al*. reported that the administration of polysaccharides from *Cyclina sinensis* significantly decreased the serum ALT and AST levels in the carbon tetrachloride -induced hepatocyte toxicity model in mice^43^.

### Cytokines in LPS-treated mice

**Figure 6:**
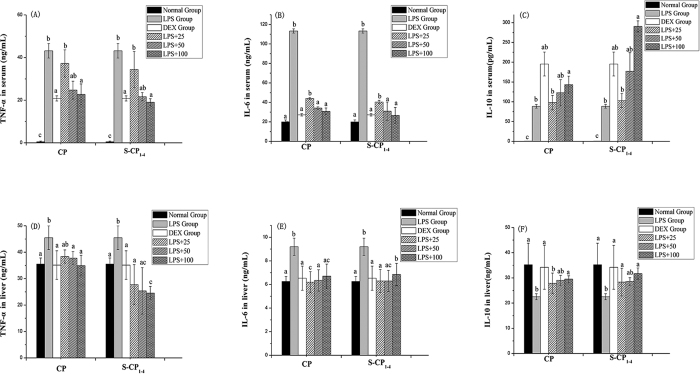
Effect of CP and S-CP_1–4_ on the levels of cytokines in LPS-induced mice; (**A**) The inhibitory activity of CP and S-CP_1–4_ for TNF-α in serum; (**B**) The inhibitory activity of CP and S-CP_1–4_ for IL-6 in serum; (**C**) The inhibitory activity of CP and S-CP_1–4_ for IL-10 in serum; (**D**) The inhibitory activity of CP and S-CP_1–4_ for TNF-α in homogenate; (**E**) The inhibitory activity of CP and S-CP_1–4_ for IL-6 in homogenate; (**F**) The inhibitory activity of CP and S-CP_1–4_ for IL-10 in homogenate. Values are expressed as mean ± SD (n = 6). Data marked withdifferent letters (a–d) differ significantly (*P* < *0.05*).

To study the protective effect of polysaccharides on LPS-treated mice, the cytokine levels were measured in the serum and liver homogenate. As shown in Fig. 6A and B, treatment with LPS significantly increased the levels of pro-inflammatory mediators in serum, including TNF-α and IL-6, but reduced the level of IL-10, one of the anti-inflammatory and immunosuppressive cytokines. In the CP and S-CP_1–4_ groups, the levels of TNF-α and IL-6 were decreased but the level of IL-10 was increased (Fig. 6C). The treatment of S-CP_1–4_ at 25 mg/mL increased the production of IL-10, indicating the good anti-inflammatory activity of the sulfated polysaccharide. However, TNF-α and IL-6 were pro-inflammatory mediators, the inhibition effects of CP and S-CP_1–4_ on TNF-α and IL-6 increased with the increasing sample concentrations. At a concentration of 100 mg/mL, the inhibitory effect of S-CP_1–4_ was dramatically improved as compared with the CP group. At all concentrations, the effect of S-CP_1–4_ was stronger than that of the CP group. Apparently, the results demonstrated that the administration of sulfated modification could improve the immunological competence of LPS-treated mice.

To determine whether CP and S-CP_1–4_ can affect the immunological functions of the liver, the production of cytokines was also assessed. The levels of TNF-α and IL-6 in the liver homogenate were significantly higher in the LPS group than in the normal group (Fig. 6). The supplementation of CP and S-CP_1–4_ significantly suppressed the increase in the level of TNF-α or IL-6 in the liver homogenate (*P* < *0.05*). The IL-10 contents were lower in the LPS group than in the normal group. The CP and S-CP_1–4_ supplementation significantly increased the IL-10 level (*P* < *0.05*).

Cytokines are important indicators of hepatocyte damage. It was reported that polysaccharides could significantly inhibit pro-inflammatory mediators, such as TNF-α, IL-1β and IL-6^44^. In this study, the levels of TNF-α and IL-6 in serum and homogenate of the model group were significantly higher than those in the normal control group. However, the up-regulation of these inflammatory factors was markedly inhibited by treatment with CP and S-CP_1–4_ (25, 50 and 100 mg/kg); S-CP_1–4_ had a stronger inhibitory activity than the native polysaccharides. Therefore, CP may exert a therapeutic effect, possibly through the restriction of pro-inflammatory mediators, and sulfated modification improved this activity.

### SOD activity, T-AOC and MDA levels in LPS-treated mice

**Figure 7:**
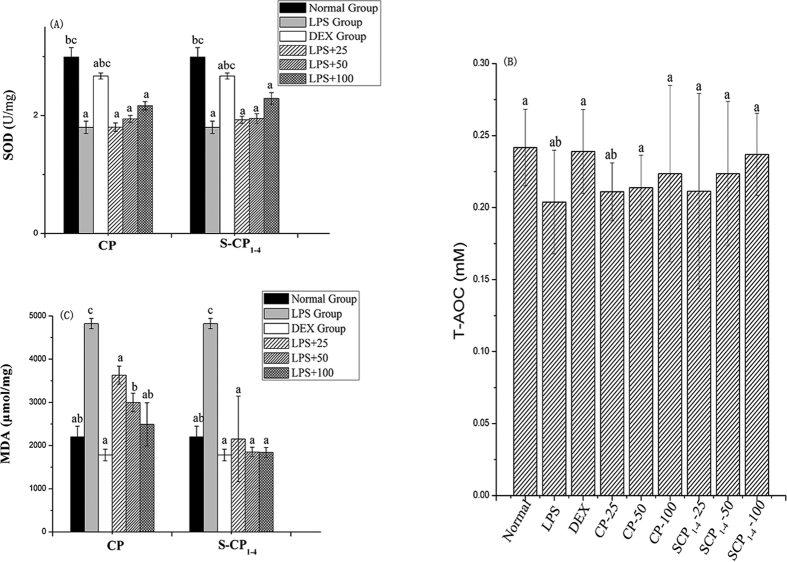
Effect of CP and S-CP_1–4_ on SOD activity (**A**) T-AOC (**B**) and MDA (**C**) levels in LPS-induced mice. Data marked withdifferent letters (a–d) differ significantly (*P* < *0.05*).

To elucidate the protective mechanisms of CP and S-CP_1–4_ on liver damage induced by LPS, SOD activity, and MDA and T-AOC levels in the liver were further studied. As shown in Fig. 7, the SOD activity and T-AOC level were markedly decreased, but the MDA level was increased in the LPS group. Therefore, LPS could cause oxidative damage in the mouse liver. The SOD activity and T-AOC content in the liver homogenate were increased by treatments with CP and S-CP_1–4_ in a dose-dependent manner. The administration of CP and S-CP_1–4_ obviously improved the hepatic SOD activity and T-AOC level but decreased the MDA level. The hepatoprotective effect of *Zizyphus jujube* polysaccharides in acetaminophen-induced liver damage was by enhancing SOD and GSH-Px activities and decreasing the level of MDA^45^. In our study, CP and S-CP_1–4_ had liver-protective effects because of their significant antioxidant activity in the body. Compared with CP, S-CP_1–4_ had a better protective effect against oxidative damage. The results showed that sulfation changed the molecular weight and physicochemical properties of polysaccharides, thereby affecting its biological activity. These results agreed with the anti-oxidative and hepatoprotective activities reported in sulfated *Codonopsis pilosula* polysaccharide. Compared with unmodified polysaccharides, the activities of ALT and AST and TNF-α level in serum and MDA level in liver homogenate of sulfated *Codonopsis pilosula* polysaccharides group were significantly lower^20^.

**Figure 8:**
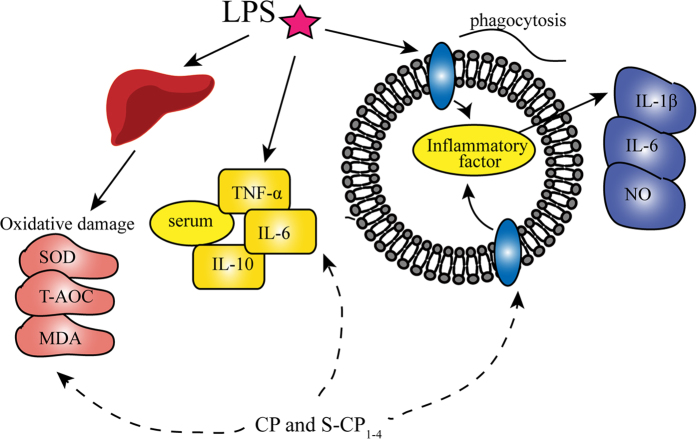
The possible anti-inflammatory mechanism of CP and S-CP_1–4_.

The fast-paced life style and environmental deterioration in modern society increase the risk of chronic diseases. The emergence of several sub-health states is often accompanied by inflammation. In this study, we found that sulfated modification of CP may enhance its anti-inflammatory activity. Given the differences caused by the sulfate group, the polysaccharide structure, including the total sugar, protein, and uronic acid content, as well as *Mw*, may result in different bioactivities. After sulfated modification, its *Mw* decreased. The stronger anti-inflammatory activity may be associated with the lower *Mw*. Meanwhile, polysaccharides have an immunomodulatory effect and antioxidant activity. CP has been found to have free radical-scavenging activity in the intestine. This study further determined its anti-inflammatory effect; as summarized in Fig. 8, the anti-inflammatory activity of polysaccharides is promoted by several factors, including the inhibitory effect of the pro-inflammatory factor, immunomodulation and anti-oxidation.

## Conclusions

The degree of substitution, *Mw*, and chemical composition of CP and S-CP_1–4_ were measured in the present study. We investigated the impact of sulfated modification on its anti-inflammatory activity *in vitro* and *in vivo*. In the LPS-induced RAW 264.7 cells, S-CP_1–4_ attenuated inflammatory mediators by inhibiting the phagocytosis of macrophages, NO production, and the release of IL-6 and IL-1β. In the LPS-injected liver damage model, liver function was significantly reduced in mice, indicating the occurrence of acute inflammation. S-CP_1–4_ and CP treatment decreased ALT activity in the serum and improved the thymus and spleen indexes. They also increased the SOD activity and T-AOC level while decreasing the MDA level. Combined with previous structural information, this effect could be attributed to the change in chemical components*, Mw*, and the degree of substitution caused by sulfated modification. In summary, sulfated modification enhanced anti-inflammatory effect of polysaccharides, and had potential value to develop non-toxic and cheap health food supplements and pharmaceuticals.

## Materials and Methods

### Plant materials

The leaves of *C. paliurus* were obtained from Jiangxi Xiushui Miraculous Tea Industry Co. (Jiangxi, China). All the leaves were air dried and stored in a cool, dark place before extraction.

### Chemicals

The T-series dextrans of different *Mw* were purchased from Pharmacia Biotech (Uppsala, Sweden). Chlorosulfonic acid, pyridine, LPS (from *Escherichia coli* 055:B5), and dexamethasone (DEX) were obtained from Sigma-Aldrich (Shanghai, China). The SOD, MDA and bicinchoninic acid (BCA) assay kits were obtained from Beyotime Institute of Biotechnology (Shanghai, China). All other chemicals were analytical grade. Aqueous solutions were prepared with ultra-pure water from a Milli-Q water purification system (Millipore, Bedford, MA, USA).

### Preparation of CP and S-CP_1–4_

CP was isolated as previously described^24^. Briefly, the leaves of *C. paliurus* were crushed and soaked in ethyl alcohol to remove the lipid and pigment. After ethyl alcohol was volatized, the residues were extracted at 90 °C with a 10-fold volume of distilled water for 2 h. The mixture was filtered with Whatman Grade No. 1 paper, and the precipitate was collected for another round of extraction. The supernatants were concentrated in a vacuum at 60 °C and precipitated with 95% ethanol (1:5, *v/v*) for 24 h at 4 °C. The precipitates were dissolved in ultrapure water and deproteinized by the Sevag method. The supernatant was collected and dialyzed to remove impurities. The resulting solution was lyophilized to yield CP.

S-CP_1–4_ was prepared according to our previous study^46^. Briefly, a known volume of pyridine was placed in an ice water bath with agitation. Chlorosulfonic acid was dripped into the anhydrous pyridine at ratio of 1:4 (*v:v*) to obtain the sulfated reagent. CP (600 mg) was suspended in formamide (20 mL) before the sulfated reagent (5 mL) was added dropwise. The mixture was allowed to react for 4 h at 60 °C. After termination of the reaction, the reactant was cooled to room temperature. The pH was adjusted to 7.0 with 4 moL/L sodium hydroxide. The resulting supernatant was collected by centrifugation, precipitated with alcohol, dialyzed, and freeze-dried to obtain S-CP_1–4_.

### Determination of total sugar, protein, uronic acid and sulfated group contents

The chemical compositions of CP and S-CP_1–4_ were determined by spectrophotometry. The total sugar content was measured by the phenol–sulfuric acid method, with glucose as the standard^47^. The protein content was determined by the Coomassie brilliant blue method, with bovine serum albumin as the standard^48^. The uronic acid content was determined by the carbazole and sulfuric acid method, with galacturonic acid as the standard^49^. The degree of sulfated polysaccharides was measured by the barium chloride–gelatin method^50^.

### Homogeneity and *Mw* determination

The homogeneity and *Mw* were analyzed by high-performance gel permeation chromatography (HPGPC) with a Waters Ultrahydrogel-500 column (7.8 mm × 300 mm)^51^. The mobile phase was ultrapure water with 0.02% NaN_3_ at a flow rate of 0.6 mL/min. The sample was dissolved in the mobile phase for 1 mg/mL, and the injection volume was 20 μL. A standard curve was drawn with glucose and the standard dextrans T-10, T-20, T-40, T-50, T-70, T-200, T-500 and T-2000 according to the time of the maximum peak and the logarithm of their respective *Mw*.

### Fourier-transform infrared spectroscopy (FT-IR)

The FT-IR spectra of purified polysaccharides were recorded in the range of 4000–400 cm^−1^ with a Thermo Nicolet 5700 FT-IR spectrophotometer (Thermo Electron, Madison, WI, USA). The polysaccharides were mixed with KBr powder at 1:100 to form pellets^52^.

### Anti-inflammatory activity of CP and S-CP_1–4_ in LPS-treated RAW 264.7 cells

#### Cell culture and experimental design

The murine macrophage cell line RAW 264.7 was purchased from the Cell Bank of the Chinese Academy of Sciences (Shanghai, China). The RAW 264.7 cells were cultured in DMEM supplemented with 10% fetal bovine serum and 2 mM l-glutamine. The cells were incubated at 37 °C in an atmosphere containing 5% CO_2_ for further study.

RAW 264.7 cells were seeded in 96-well or 6-well plates at a density of 1 × 10^5^ cells/mL and cultivated for 4 h in an incubator under a 5% CO_2_ atmosphere at 37 °C. The medium was carefully removed and different concentrations of CP and S-CP_1–4_ were added (25, 50, and 100 μg/mL). Simultaneously, 1 μg/mL DEX was added in an equal volume as the positive control. After 24 h of incubation, LPS was added at a concentration of 1 μg/mL for 12 h in the LPS, CP, S-CP_1–4_, and DEX groups. Meanwhile, an isopycnic culture medium was placed in the control group. The supernatant in 6-well plates was collected to study the cytokine secretion and nitrite production, whereas the 96-well plates were used to measure phagocytic activity.

#### Cell viability

RAW264.7 cells were seeded in 96-wells plates cultivated as mentioned above, and the cell viability was measured using the CCK-8 assay^53^. After attachment culture for 4 h, the culture medium was removed and the cells were washed with phosphate buffered saline (PBS). The cells were exposed to different concentrations of the CP and S-CP_1–4_, along with the normal group. After 24 h incubation, the cells in each well were treated with 10 μL CCK-8 solution and incubated in an atmosphere of 5% CO_2_ at 37 °C for 2 h. The absorption values were measured at 570 nm, using a spectrophotometer (Varioskan Flash Multimode Reader, Thermo Fisher Scientific, USA).

#### Cytokine secretion, nitrite production and phagocytic activity

RAW 264.7 cells were treated as mentioned above; the NO levels were measured by nitrate reductase with a NO assay kit (Nanjing Jiancheng Bioengineering Institute, China)^54^. The levels of IL-1β and IL-6 secretion in the culture medium were determined with an ELISA kit (Boster, China)^55^. The phagocytic ability of macrophages was measured based on the neutral red uptake. The optical density at 540 nm was measured with an automatic microplate reader (Themo, Waltham, USA)^56^.

### Anti-inflammatory activity of CP and S-CP_1–4_ in LPS-treated mice

#### Animals and experimental design

A total of 90 male mice weighing 18–20 g were purchased from the Hunan Slaccas Laboratory Animal Company and used for experiments [Certificate Number SCXK(xiang)2011–0003, Hunan, China]. Before the experiments, all the animals were housed at 25 ± 2 °C with 12 h light–dark cycles for at least 7 d before the experiments. Mice were maintained and cared for in compliance with the Guidelines for the Care and Use of Laboratory Animals published by the U.S. National Institutes of Health (NIH Publication 85–23, 1996). All entire experimental procedures were conducted according to the protocols approved by Animal Care and Use Committee of Nanchang University.

The mice were randomly divided into 9 groups (n = 10): the normal group, the LPS group, the CP (25, 50 and 100 mg/kg BW) groups, the S-CP_1–4_ (25, 50 and 100 mg/kg) groups, and the DEX group. The CP and S-CP_1–4_ treatment groups received intragastric administration at various doses; the DEX group was given 5 mg/kg DEX. The normal and LPS groups were given a gavage of normal saline at the same volume. On the seventh day of the experiment, all mice were fasted for 12 h. Except for the control group, all groups were intraperitoneally injected with LPS (0.5 mg/kg). At 2 h after induction, mice were sacrificed to collect the serum; the plasma and tissue samples were then stored at −80 °C.

#### Measurement of visceral index and liver function

The thymus, spleen, and liver samples were collected under anaesthetized conditions to calculate the visceral index. Liver function was evaluated by the activities of AST and ALT in serum with commercial kits (Nanjing Jiancheng Bioengineering Institute, China)^57^,^58^.

#### Measurement of SOD activity, and MDA and T-AOC levels in Liver

SOD activity, and MDA and T-AOC levels in the liver tissue homogenate were measured using commercial kits (Beyotime Institute of Biotechnology, Shanghai, China)^59^,^60^.

#### ELISA assays for TNF-α, IL-6 and IL-10 levels

TNF-α, IL-6 and IL-10 levels in serum and liver homogenate were determined by enzyme linked immunosorbent assay kits purchased from Wuhan Boster Biological Engineering Co.(Wuhan, Hubei, China)^61^,^62^.

### Statistical analysis

Results were expressed as means ± SD. The statistical significance of any difference in each parameter among the groups was evaluated by one-way ANOVA, followed by Duncan’s multiple range test using SPSS 11.0. P values of <0.05 were considered statistically significant.

## Additional Information

**How to cite this article**: Wang, Z. *et al*. Sulfated *Cyclocarya paliurus* polysaccharides markedly attenuates inflammation and oxidative damage in lipopolysaccharide-treated macrophage cells and mice. *Sci. Rep.* **7**, 40402; doi: 10.1038/srep40402 (2017).

**Publisher's note:** Springer Nature remains neutral with regard to jurisdictional claims in published maps and institutional affiliations.

## Figures and Tables

**Table 1:** The chemical composition and molecular weight of CP and S-CP_1–4_.

	CP	S-CP_1–4_
Carbohydrate (%)	63.77 ± 1.55	42.41 ± 2.55
Uronic acid (%)	13.41 ± 0.69	11.03 ± 0.17
Protein (%)	8.23 ± 0.78	1.95±0.09
Degree of sulfated group	–nd	0.42 ± 0.04
Molecular weight (Da)	1.39 × 10^5^	2.12 × 10^5^

^A^Values were expressed as mean ± SD and three replicated independent determinations.

^B^–nd, not detected.

## References

[b1] Kolaczkowska E. & Kubes P. Neutrophil recruitment and function in health and inflammation. Nat Rev Immunol. 13, 159–175 (2013). 23435331 10.1038/nri3399

[b2] Strowig T., Henao Mejia J., Elinav E. & Flavell R. Inflammasomes in health and disease. Nature. 481, 278–286 (2012). 22258606 10.1038/nature10759

[b3] Tracy R. Emerging relationships of inflammation, cardiovascular disease and chronic diseases of aging. Int J Obes Relat Metab Disord. 27 (2003). 10.1038/sj.ijo.0802497 14704741

[b4] Coussens L. M. & Werb Z. Inflammation and cancer. Nature. 420, 860–867 (2002). 12490959 10.1038/nature01322 PMC2803035

[b5] Ferguson L. R. & Schlothauer R. C. The potential role of nutritional genomics tools in validating high health foods for cancer control: Broccoli as example. Mol. Nutr. Food Res. 56, 126–146 (2012). 22147677 10.1002/mnfr.201100507

[b6] Ferreira S. S., Passos C. P., Madureira P., Vilanova M. & Coimbra M. A. Structure–function relationships of immunostimulatory polysaccharides: A review. Carbohydr Polym. 132, 378–396 (2015). 26256362 10.1016/j.carbpol.2015.05.079

[b7] Raetz C. R. & Whitfield C. Lipopolysaccharide endotoxins. Annu Rev Biochem. 71, 635–700 (2002). 12045108 10.1146/annurev.biochem.71.110601.135414 PMC2569852

[b8] Liao C. H., Guo S. J. & Lin J. Y. Characterisation of the chemical composition and *in vitro* anti-inflammation assessment of a novel lotus (*Nelumbo nucifera Gaertn*) plumule polysaccharide. Food Chem. 125, 930–935 (2011).

[b9] Hwang P. A. . Inhibition of lipopolysaccharide (LPS)-induced inflammatory responses by *Sargassum hemiphyllum* sulfated polysaccharide extract in RAW 264.7 macrophage cells. J Agric Food Chem. 59, 2062–2068 (2011). 21322561 10.1021/jf1043647

[b10] Du Y. Q., Liu Y. & Wang J. H. Polysaccharides from *Umbilicaria esculenta* cultivated in Huangshan Mountain and immunomodulatory activity. Int J Biol Macromol. 72, 1272–1276 (2015). 25316425 10.1016/j.ijbiomac.2014.09.057

[b11] Pan L. H. . Comparison of hypoglycemic and antioxidative effects of polysaccharides from four different *Dendrobium* species. Int J Biol Macromol. 64, 420–427 (2014). 24370475 10.1016/j.ijbiomac.2013.12.024

[b12] Seibel J., Molzberger A. F., Hertrampf T., Laudenbach-Leschowski U. & Diel P. Oral treatment with genistein reduces the expression of molecular and biochemical markers of inflammation in a rat model of chronic TNBS-induced colitis. Eur J Nutr. 48, 213–220 (2009). 19234664 10.1007/s00394-009-0004-3

[b13] Chai Y. Y., Wang G. B., Fan L. L. & Zhao M. A proteomic analysis of mushroom polysaccharide-treated HepG2 cells. Sci Rep. 6, 23565, doi: 10.1038/srep23565 (2016). 27020667 PMC4810362

[b14] Tan W. C. . *Ganoderma* neo-japonicum Imazeki revisited: Domestication study and antioxidant properties of its basidiocarps and mycelia. Sci Rep. 5, 12515, doi: 10.1038/srep12515 (2015). 26213331 PMC4515590

[b15] Zha X. Q. . Molecular mechanism of a new *Laminaria japonica* polysaccharide on the suppression of macrophage foam cell formation via regulating cellular lipid metabolism and suppressing cellular inflammation. Mol. Nutr. Food Res. 59, 2008–2021 (2015). 26153221 10.1002/mnfr.201500113

[b16] Xiao J. . *Lycium barbarum* polysaccharides therapeutically improve hepatic functions in non-alcoholic steatohepatitis rats and cellular steatosis model. Sci Rep. 4, 5587. doi: 10.1038/srep05587 (2014). 24998389 PMC4083265

[b17] Chang C. W., Lur H. S., Lu M. K. & Cheng J. J. Sulfated polysaccharides of *Armillariella mellea* and their anti-inflammatory activities via NF-κB suppression. Food Res Int. 54, 239–245 (2013).

[b18] Chen Y. . Sulfated modification of the polysaccharides from *Ganoderma* atrum and their antioxidant and immunomodulating activities. Food Chem. 186, 231–238 (2015). 25976815 10.1016/j.foodchem.2014.10.032

[b19] Cheng J. J., Chao C. H., Chang P. C. & Lu M. K. Studies on anti-inflammatory activity of sulfated polysaccharides from cultivated *fungi Antrodia cinnamomea*. Food Hydrocoll. 53, 37–45 (2016).

[b20] Liu C. . The comparison of antioxidative and hepatoprotective activities of *Codonopsis pilosula* polysaccharide (CP) and sulfated CP. Int Immunopharmacol. 24, 299–305 (2015). 25543057 10.1016/j.intimp.2014.12.023

[b21] Xie J. H. . Simultaneous analysis of 18 mineral elements in *Cyclocarya paliurus* polysaccharide by ICP-AES. Carbohydr Polym. 94, 216–220 (2013). 23544531 10.1016/j.carbpol.2012.12.072

[b22] National Health and Family Planning Commission of PRC http://www.nhfpc.gov.cn/ (2013).

[b23] Xie J. H. . Extraction, chemical composition and antioxidant activity of flavonoids from *Cyclocarya paliurus* (Batal.) Iljinskaja leaves. Food Chem. 186, 97–105 (2015). 25976797 10.1016/j.foodchem.2014.06.106

[b24] Xie J. H. . Isolation, chemical composition and antioxidant activities of a water-soluble polysaccharide from *Cyclocarya paliurus* (Batal.) Iljinskaja. Food Chem. 119, 1626–1632 (2010).

[b25] Xie J. H. . Purification, physicochemical characterisation and anticancer activity of a polysaccharide from *Cyclocarya paliurus* leaves. Food Chem. 136, 1453–1460 (2013). 23194548 10.1016/j.foodchem.2012.09.078

[b26] Min F. F., Wan Y. J., Nie S. P. & Xie M. Y. Study on colon health benefit of polysaccharide from *Cyclocarya paliurus* leaves *in vivo*. J Funct Foods. 11, 203–209 (2014).

[b27] Liu Y. H. . Sulfation of a polysaccharide obtained from *Phellinus ribis* and potential biological activities of the sulfated derivatives. Carbohydr Polym. 77, 370–375 (2009).

[b28] Wang J. L. . Sulfated modification, characterization and structure–antioxidant relationships of *Artemisia sphaerocephala* polysaccharides. Carbohydr Polym. 81, 897–905 (2010).

[b29] Chen H. X., Zhang M., Qu Z. S. & Xie B. J. Antioxidant activities of different fractions of polysaccharide conjugates from green tea (*Camellia Sinensis*). Food Chem. 106, 559–563 (2008).

[b30] Wang Y. F., Mao F. F. & Wei X. L. Characterization and antioxidant activities of polysaccharides from leaves, flowers and seeds of green tea. Carbohydr Polym. 88, 146–153 (2012).

[b31] Xie J. H., Tang W., Jin M. L., Li J. E. & Xie M. Y. Recent advances in bioactive polysaccharides from *Lycium barbarum* L., *Zizyphus jujuba* Mill, *Plantago* spp., and *Morus* spp.: Structures and functionalities. Food Hydrocoll. 60, 148–160 (2016).

[b32] Zhang H. . Structural characterisation of a novel bioactive polysaccharide from *Ganoderma atrum*. Carbohydr Polym. 88, 1047–1054 (2012). 10.1016/j.carbpol.2016.11.088 28024543

[b33] Sun Y. X. . Sulfated modification of the water-soluble polysaccharides from *Polyporus albicans mycelia* and its potential biological activities. Int J Biol Macromol. 44, 14–17 (2009). 18951914 10.1016/j.ijbiomac.2008.09.010

[b34] Xu Y. F. . Sulfated modification of the polysaccharide from *Sphallerocarpus gracilis* and its antioxidant activities. Int J Biol Macromol. 87, 180–190 (2016). 26893048 10.1016/j.ijbiomac.2016.02.037

[b35] Li X. L. . Structural identification and sulfated modification of an antiglycation *Dendrobium huoshanense* polysaccharide. Carbohydr Polym. 106, 247–254 (2014). 24721075 10.1016/j.carbpol.2014.02.029

[b36] Förstermann U. & Sessa W. C. Nitric oxide synthases: regulation and function. Eur Heart J. 33, 829–837 (2012). 21890489 10.1093/eurheartj/ehr304 PMC3345541

[b37] Tabarsa M. . Structure-activity relationships of sulfated glycoproteins from *Codium fragile* on nitric oxide releasing capacity from RAW264. 7 cells. Mar Biotechnol. 17, 266–276 (2015). 25627693 10.1007/s10126-015-9615-2

[b38] Luo T. . Astragalus polysaccharide attenuates lipopolysaccharide-induced inflammatory responses in microglial cells: regulation of protein kinase B and nuclear factor-κB signaling. Inflamm Res. 64, 205–212 (2015). 25669325 10.1007/s00011-015-0798-9

[b39] Wang X. F. . Sulfated *Astragalus* polysaccharide can regulate the inflammatory reaction induced by LPS in Caco2 cells. Int J Biol Macromol. 60, 248–252 (2013). 23751319 10.1016/j.ijbiomac.2013.05.037

[b40] Zhu L. N. . Isolation and purification of a polysaccharide from the caterpillar medicinal mushroom *Cordyceps militaris* (Ascomycetes) fruit bodies and its immunomodulation of RAW 264.7 macrophages. Int J Med Mushrooms. 16, 247–257 (2014). 24941166 10.1615/intjmedmushr.v16.i3.50

[b41] Dore C. M. P. G. . A sulfated polysaccharide, fucans, isolated from brown algae *Sargassum vulgare* with anticoagulant, antithrombotic, antioxidant and anti-inflammatory effects. Carbohydr Polym. 91, 467–475 (2013). 23044157 10.1016/j.carbpol.2012.07.075

[b42] Wang J. M. . Sulfated modification can enhance the immune-enhancing activity of *lycium barbarum* polysaccharides. Cell Immunol. 263, 219–223 (2010). 20434140 10.1016/j.cellimm.2010.04.001

[b43] Jiang C. X. . Antioxidant activity and potential hepatoprotective effect of polysaccharides from *Cyclina sinensis*. Carbohydr Polym. 91, 262–268 (2013). 23044131 10.1016/j.carbpol.2012.08.029

[b44] Xie J. H. . Advances on bioactive polysaccharides from medicinal plants. Crit Rev Food Sci. 56, S60–S84 (2016). 10.1080/10408398.2015.1069255 26463231

[b45] Liu G. P. . Hepatoprotective effects of polysaccharides extracted from *Zizyphus jujube* cv. Huanghetanzao. Int J Biol Macromol. 76, 169–175 (2015). 25709018 10.1016/j.ijbiomac.2015.01.061

[b46] Xie J. H. . Sulfated modification, characterization and antioxidant activities of polysaccharide from *Cyclocarya paliurus*. Food Hydrocoll. 53, 7–15 (2016).

[b47] Dubois M., Gilles K. A., Hamilton J. K., Rebers P. & Smith F. Colorimetric method for determination of sugars and related substances. Anal Chem. 28, 350–356 (1956).

[b48] Sedmak J. J. & Grossberg S. E. A rapid, sensitive, and versatile assay for protein using Coomassie brilliant blue G250. Anal Biochem. 79, 544–552 (1977). 68686 10.1016/0003-2697(77)90428-6

[b49] Selvendran R. R., March J. F. & Ring S. G. Determination of aldoses and uronic acid content of vegetable fiber. Anal. Biochem. 96, 282–292 (1979). 474957 10.1016/0003-2697(79)90583-9

[b50] Lu Y., Wang D. Y., Hu Y. L., Huang X. Y. & Wang J. M. Sulfated modification of *epimedium* polysaccharide and effects of the modifiers on cellular infectivity of IBDV. Carbohydr Polym. 71, 180–186 (2008).

[b51] Chen Y., Xie M. Y., Nie S. P., Li C. & Wang Y. X. Purification, composition analysis and antioxidant activity of a polysaccharide from the fruiting bodies of *Ganoderma atrum*. Food Chem. 107, 231–241 (2008).

[b52] Kačuráková M. . Characterisation of xylan-type polysaccharides and associated cell wall components by FT-IR and FT-Raman spectroscopies. Food Hydrocoll. 13, 35–41 (1999).

[b53] Wang Z. J. . Sulfated polysaccharides from *Cyclocarya paliurus* reduce H_2_O_2_-induced oxidative stress in RAW264.7 cells. Int J Biol Macromol. 80, 410–417 (2015). 26111910 10.1016/j.ijbiomac.2015.06.031

[b54] Zhang L. . Immunomodulatory activities of polysaccharides isolated from *Taxillus chinensis* and *Uncaria rhyncophylla*. Carbohydr Polym. 98, 1458–1465 (2013). 24053827 10.1016/j.carbpol.2013.07.060

[b55] Yu Q. . Macrophage immunomodulatory activity of a purified polysaccharide isolated from *Ganoderma atrum*. Phytother Res. 27, 186–191 (2013). 22511240 10.1002/ptr.4698

[b56] Du H. T. . Extraction optimization, preliminary characterization and immunological activities *in vitro* of polysaccharides from *Elaeagnus angustifolia* L. pulp. Carbohydr Polym. 151, 348–357 (2016). 27474576 10.1016/j.carbpol.2016.05.068

[b57] Yang X. B., Yang S., Guo Y. R., Jiao Y. D. & Zhao Y. Compositional characterisation of soluble apple polysaccharides, and their antioxidant and hepatoprotective effects on acute CCl _4_-caused liver damage in mice. Food Chem. 138, 1256–1264 (2013). 23411241 10.1016/j.foodchem.2012.10.030

[b58] Zhao T. . Antitumor and immunomodulatory activity of a water-soluble low molecular weight polysaccharide from *Schisandra chinensis* (Turcz.) Baill. Food Chem Toxicol. 55, 609–616 (2013). 23416131 10.1016/j.fct.2013.01.041

[b59] Cui H. X., Li T., Wang L. P., Su Y. & Xian C. J. *Dioscorea bulbifera* polysaccharide and cyclophosphamide combination enhances anti-cervical cancer effect and attenuates immunosuppression and oxidative stress in mice. Sci Rep. 5, 19185, doi: 10.1038/srep19185 (2016). 26753518 PMC4709656

[b60] Liu H. . Protective effects of sea *buckthorn* polysaccharide extracts against LPS/d-GalN-induced acute liver failure in mice via suppressing TLR4-NF-κB signaling. J Ethnopharmacol. 176, 69–78 (2015). 26494508 10.1016/j.jep.2015.10.029

[b61] Cao Y. Z. . Protective effect of Ulinastatin against murine models of sepsis: inhibition of TNF-α and IL-6 and augmentation of IL-10 and IL-13. Exp Toxicol Pathol. 64, 543–547 (2012). 21159497 10.1016/j.etp.2010.11.011

[b62] Piccioni M. . A purified *capsular* polysaccharide markedly inhibits inflammatory response during endotoxic shock. Infect Immun. 81, 90–98 (2013). 23090956 10.1128/IAI.00553-12 PMC3536145

